# Human Gingival Fibroblasts Exposed to Extremely Low-Frequency Electromagnetic Fields: In Vitro Model of Wound-Healing Improvement

**DOI:** 10.3390/ijms20092108

**Published:** 2019-04-29

**Authors:** Erica Costantini, Bruna Sinjari, Chiara D’Angelo, Giovanna Murmura, Marcella Reale, Sergio Caputi

**Affiliations:** Department of Medical, Oral and Biotechnological Science, University “G. d’Annunzio” Chieti-Pescara, 66100 Chieti, Italy; b.sinjari@unich.it (B.S.); chiara.dangelo@unich.it (C.D.); g.murmura@unich.it (G.M.); sergio.caputi@unich.it (S.C.)

**Keywords:** electromagnetic fields, human gingival fibroblasts, cytokines, chemokines

## Abstract

Several clinical studies have suggested the impact of sinusoidal and pulsed electromagnetic fields in quickening wound repair processes and tissue regeneration. The clinical use of extremely low-frequency electromagnetic fields could represent a novel frontier in tissue repair and oral health, with an interesting clinical perspective. The present study aimed to evaluate the effect of an extremely low-frequency sinusoidal electromagnetic field (SEMF) and an extremely low-frequency pulsed electromagnetic field (PEMF) with flux densities of 1 mT on a model of oral healing process using gingival fibroblasts. An in vitro mechanical injury was produced to evaluate wound healing, migration, viability, metabolism, and the expression of selected cytokines and protease genes in fibroblasts exposed to or not exposed to the SEMF and the PEMF. Interleukin 6 (IL-6), transforming growth factor beta 1 (TGF-β), metalloproteinase 2 (MMP-2), monocyte chemoattractant protein 1 (MCP-1), inducible nitric oxide synthase (iNOS), and heme oxygenase 1 (HO-1) are involved in wound healing and tissue regeneration, favoring fibroblast proliferation, chemotaxis, and activation. Our results show that the exposure to each type of electromagnetic field increases the early expression of IL-6, TGF-β, and iNOS, driving a shift from an inflammatory to a proliferative phase of wound repair. Additionally, a later induction of MMP-2, MCP-1, and HO-1 was observed after electromagnetic field exposure, which quickened the wound-healing process. Moreover, electromagnetic field exposure influenced the proliferation, migration, and metabolism of human gingival fibroblasts compared to sham-exposed cells. This study suggests that exposure to SEMF and PEMF could be an interesting new non-invasive treatment option for wound healing. However, additional studies are needed to elucidate the best exposure conditions to provide the desired in vivo treatment efficacy.

## 1. Introduction

In the last few years, many studies have focused on the interaction between non-ionizing electromagnetic fields and living organisms, considering that the geomagnetic field is one of the physical environmental factors to which biological systems are exposed [1], and have highlighted the negative or positive impact of exposure to electromagnetic fields (EMFs) [2]. EMFs can be defined as low frequency (LF, range: 30–300 kHz) or extremely low frequency (ELF, range: 3–30 Hz). 

The biological effects of electromagnetic fields depend on the intensity and frequency range of the employed signals, on their amplitude, waveform, polarization, and dose, and on the exposure time to the EMF. Furthermore, several studies have highlighted that the biological effects of EMFs are related to the types of specialized cells that are exposed [3,4,5,6].

Biological effects modulated by EMFs include, but are not limited to, cell migration, cell proliferation and differentiation, cytokine modulation, the expression of growth factors, and nitric oxide signaling [7,8,9,10,11,12]. Potential negative effects of ELF-EMFs have been shown in earlier studies, which demonstrated an increased risk of disease promotion and progression in childhood cancer, breast cancer, neoplastic development, neurodegenerative diseases, and in fertility and cardiovascular disorders [13,14,15,16]. However, these experimental studies did not provide convincing evidence for a relationship between exposure to ELF-EMF and disease development. 

A wide number of results have demonstrated that exposure to ELF-EMF positively modifies cell proliferation, the production of anti-inflammatory cytokines, and tissue regeneration [8,17,18]. Interestingly, there is substantial evidence that ELF-EMFs can have health-promoting effects on living organisms. Studies on the clinical applications of ELF-EMFs have reported protection against hypoxia and myocardial damage, and increased survival following ischemia-reperfusion [19,20]. ELF-EMFs have also been reported to be effective in enhancing osteogenesis and chondrogenesis in human skeletal stem cells/human bone marrow stromal cells (hSSCs/hBMSCs), with no documented negative effects [21,22].

In light of these results, the translational significance of the effects of ELF-EMFs with respect to their efficacy in treating a variety of conditions is of ever-increasing interest. Thus, the development of innovative EMF-based treatments and the study of the biological effects of EMFs have been explored in the field of regenerative medicine. In particular, investigating the ways in which EMF exposure could be used to control tissue regeneration and wound healing is a stimulating research field. Previous studies were conducted using EMFs with frequencies of 50/60 Hz, and with different magnetic field intensities, however not with a flux density higher than 20 mT, and consequently, these had different therapeutic efficacy [23].

One of the most interesting biological effects of ELF-EMFs is clinically effective wound repair, bone healing, and neuronal regeneration [18,24,25]. Most of the clinical studies and therapeutic applications using ELF-EMFs have been performed with pulsed electromagnetic fields (PEMFs), which have been found to be effective in reducing healing time, the rate of recurrence of venous leg ulcers [26,27], and pain, in patients with chronic ulcerations and bone fractures [28]. However, few studies have been carried out to evaluate the biological impact of sinusoidal electromagnetic fields (SEMFs) [29,30]. Moreover, although more knowledge about the effects of EMFs on wound healing is becoming available, the underlying cellular mechanisms remain poorly understood. Furthermore, the ELF-EMF waveform, intensity, and frequency, and the exposure time to ELF-EMF which result in better wound healing have not been completely determined. 

Wound healing is a complex process that arises on the skin or in the oral mucosa, and involves a series of four distinct stages: (1) hemostasis; (2) inflammation; (3) proliferation; and (4) remodeling. The hemostatic phase results from the immediate activation of platelets, which release molecules such as growth factor and cytokines that initiate and influence wound repair. Within 24 h after injury, inflammation occurs via inflammatory pathways and immune system activation in order to remove cell debris and prevent infection [31,32] by recruiting and activating different cell types, such as epithelial cells and fibroblasts, which are responsible for the production of inflammatory proteins and the regulation of gene expression [33]. The proliferation phase is primarily initiated by platelets and macrophages, with the stimulation of fibroblasts and epidermal cells, which contribute to epithelialization through the production of growth factors such as transforming growth factor beta 1 (TGF-β) and vascular endothelial growth factor (VEGF). Remodeling is the final stage of healing, in which inflammatory cells terminate their action and the release of growth factors is reduced. 

Wound healing is a protective mechanism of the body which leads to the recovery of functionality and the promotion of health [34]. In the oral cavity, there are millions of microorganisms that have been implicated in oral and systemic diseases. Thus, wound repair is a critical process for preventing microbes, or other agents, from invading deeper into tissues. After tooth extraction, the sequence of healing events is represented by blood coagulation, the production of chemotactic and inflammatory mediators, re-epithelialization, and bone regeneration [35,36]. In any phase of this repair process, adverse conditions that may negatively impact the healing could be present, such as alveolitis, granuloma formation, fistulae, and ulcers [37,38,39,40,41].

In our previous studies, keratinocytes were used as an in vitro model of skin mechanical injury, and exposure to a 1 mT SEMF was shown to be able to quicken a wound closure, with inflammatory response and oxidative stress regulation [42,43]. 

In this scenario, exposure to an ELF-EMF may represent a suitable solution to promote wound healing and avoid side effects. 

The aim of this study was to investigate the effects of an ELF-SEMF and an ELF-PEMF on an in vitro soft-tissue wound model obtained by mechanical injury on primary human gingival fibroblasts (hGFs). The proliferation and migration of hGFs and the expression of inflammatory mediators were analyzed after exposure to the SEMF and to the PEMF.

Our results highlight the ability of SEMF and PEMF to modulate inflammatory cytokines, even with different timing, thus favoring wound healing.

## 2. Results

### 2.1. Human Gingival Fibroblast Growth Curves

**Figure 1:**
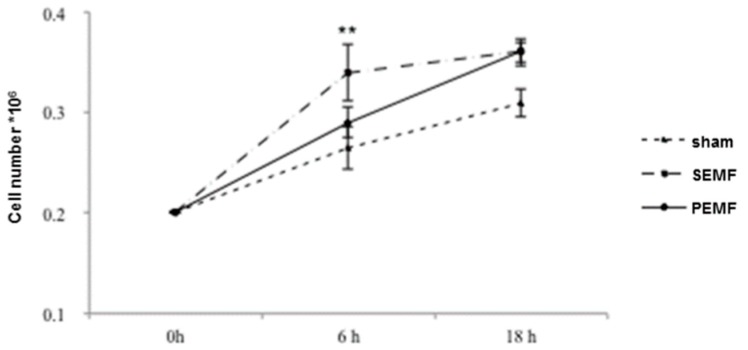
Numbers of human gingival fibroblast (hGF) cells following exposure to two types of electromagnetic field (EMF). The hGF cell numbers were calculated after 6 and 18 h exposure to a sham, a sinusoidal electromagnetic field (SEMF), and a pulsed electromagnetic field (PEMF). The means ± standard deviation (SD) of the change from baseline (0 h) over time are reported in the graph. Experiments were carried out in duplicate, and hGFs derived from the 11 recruited patients, were analyzed. ** *p*-Value < 0.01 SEMF vs. sham.

In order to evaluate the effects of an SEMF and a PEMF on the growth of hGFs, hGFs were seeded in six well plates, which were subsequently placed in the central part of a solenoid and exposed to a 1 mT PEMF, a 1 mT SEMF, or to sham, for periods of 6 and 18 h. Figure 1 shows the number of cells obtained after counting after 6 and 18 h. In the sham-exposed cells, a slow increase in cell number was observed at both counting times. However, the cell number after 6 h differed between exposure to the SEMF and the PEMF. A greater increase in cell number was detected following cell culture exposure to the SEMF (∆ = 0.075), while a lower increase was observed following exposure to the PEMF (∆ = 0.03). After 18 h exposure, the cell proliferation rate was comparable for both the SEMF- and PEMF-exposed cells (∆ = 0.05 and ∆ = 0.03, respectively). These results underline the different effects of different exposure times to SEMF and PEMF on cell proliferation, with SEMF exhibiting an earlier effect. 

### 2.2. Electromagnetic Field (EMF) and Cell Metabolic Activity

**Figure 2:**
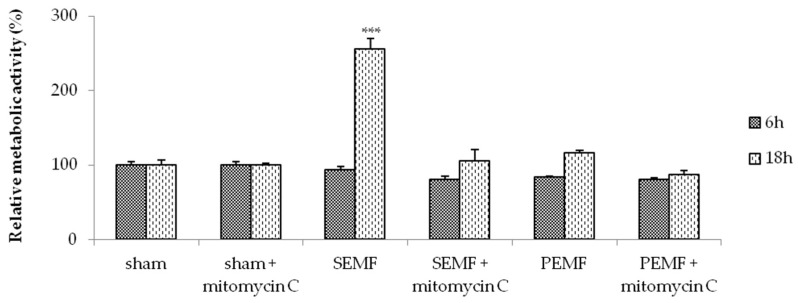
Results of the 3-(4,5-dimethylthiazol-2-yl)-2,5-diphenyl tetrazolium bromide cell-viability assay (MTT assay), showing the metabolic activity rates of hGFs, pre-treated or not with mitomycin C, exposed to sham, the SEMF, and the PEMF. Experiments were carried out in duplicate, and hGFs derived from all recruited patients were analyzed. Values are reported as the mean ± SD change with respect to sham-exposed hGFs assumed equal to 100% of activity. *** *p*-Value < 0.001 SEMF vs. sham.

We performed a 3-(4,5-dimethylthiazol-2-yl)-2,5-diphenyl tetrazolium bromide cell-viability assay (MTT assay) to verify the effects of the EMFs on cell metabolism. The ability of cells to reduce MTT provides an indication of mitochondrial integrity and activity, which is interpreted as the measure of cell viability. Time-course experiments were carried out to verify the effects of the SEMF and PEMF after 6 and 18 h of exposure (Figure 2). After 6 h of SEMF and PEMF exposure, respectively, no alteration of metabolic activity was detected in the hGFs compared to the sham-exposed hGFs. However, after 18 h of exposure to the SEMF and the PEMF, respectively, cell metabolic activity was found to be significantly increased, with a higher increase observed in the SEMF-exposed cells compared to sham-exposed cells (*p* < 0.001). To understand whether EMF exposure modulates the proliferation and/or migration of hGFs during wound healing, the exposure was conducted with and without the presence of mitomycin C, a potent DNA crosslinker and inhibitor of cell proliferation. The results showed that the pre-treatment of hGFs with mitomycin C reduced the effects of EMF exposure on metabolic activity. Taken together, these results clearly demonstrate that the SEMF and PEMF differently increased hGF metabolism, and moreover that this effect may be partially inhibited by mitomycin C. 

### 2.3. Effect of Sinusoidal Electromagnetic Field (SEMF) and Pulsed Electromagnetic Field (PEMF) on Wound Closure

**Figure 3:**
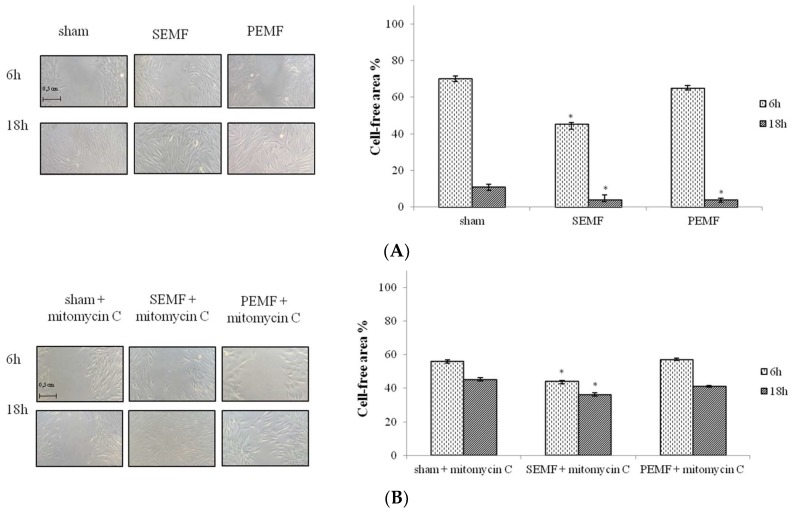
Representative light-microscope images of wound-healing assays for hGFs. (**A**) Cell migration rate was evaluated after 6 and 18 h of exposure to sham, SEMF, and PEMF. (**B**) Cell migration rate was evaluated after the exposure of mitomycin C pre-treated cells to sham, SEMF, and PEMF for 6 and 18 h. Scratch width was determined from images taken after both exposure times by measuring the distance between the two points with maximal distance on the outer edges of the cells. The average width of each scratch was calculated as a percentage of the original scratch width. The graph represents the cell migration efficiency, measured as the change in cell-free area over-time, assuming the width of the fresh wound was equal to 100%. The original microscope magnification was 10×. Scale bar = 0.5 cm. * *p*-Value ≤ 0.05 SEMF vs. sham and PEMF vs. sham.

When hGFs reached confluence, a mechanical scratch was performed on cellular monolayer. The effect on cell migration and wound closure of exposure to the 1 mT SEMF and the 1 mT PEMF for 6 and 18 h was subsequently analyzed. Quantitative evaluation performed by measuring the cell-free area showed that both the SEMF and the PEMF improved wound closure, with a higher reduction in cell-free area observed in cells exposed to the SEMF for 6 and 18 h, as shown in the representative photographs in Figure 3A. The graph in Figure 3A represents the mean percentage of cell-free area at each time point and for different exposure conditions, evaluated for hGFs isolated from all different recruited subjects. To better understand the role of cell proliferation and migration on wound healing, cells were treated with mitomycin C. Interestingly, in the presence of mitomycin C, the reduction of cell-free area was lower than that detected in its absence at both time points after exposure to both the SEMF and the PEMF, respectively. For hGFs pre-treated with mitomycin C, after 18 h of SEMF and PEMF exposure the cell-free area was lower than for sham-exposed cells both with and without mitomycin C pre-treatment (Figure 3B). These results confirm that the 1 mT SEMF and the 1 mT PEMF induce faster wound healing, by not only promoting cell migration but also cell proliferation. 

### 2.4. Effect of EMFs on the mRNA Expression

#### 2.4.1. Effect of EMFs on Interleukin 6 (IL-6), Transforming Growth Factor Beta 1 (TGF-β), and Monocyte Chemoattractant Protein 1 (MCP-1) Gene Expression

**Figure 4:**
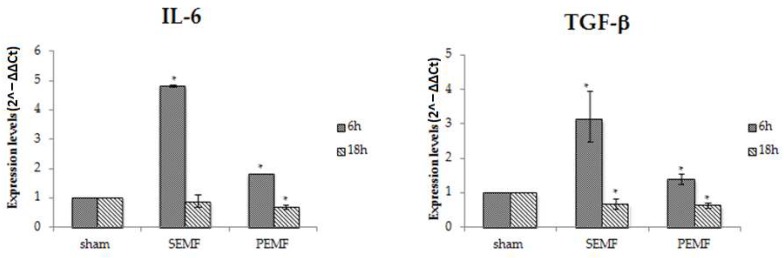
The mRNA expression of interleukin 6 (IL-6) and transforming growth factor beta 1 (TGF-β) in hGFs exposed to the SEMF and the PEMF compared to sham-exposed cells. Expression levels of IL-6 and TGF-β (2^−ΔΔ*C*t^) are reported as mean ± standard error (SE). * *p*-Value < 0.05 SEMF or PEMF vs. sham assumed as 1.

In an attempt to clarify the mechanisms by which the SEMF and the PEMF improve wound closure, an analysis of the changes in gene expression of several mediators involved in wound healing was performed. A significant increase in the expression of IL-6 and TGF-β was observed after 6 h of SEMF exposure, followed by a decrease in the expression of the same markers after 18 h, with levels comparable with those observed in sham-exposed hGFs after 18 h. When hGFs were exposed to the PEMF, a weak increase in IL-6 and TGF-β expression was observed after 6 h, while after 18 h the expression of IL-6 and TGF-β reduced to levels comparable to those detected in cells exposed to the SEMF for 18 h (Figure 4). These results show that exposure to SEMF and PEMF induces the early expression of inflammatory cytokines, driving a more precocious shift from the inflammatory to the proliferative phase of wound healing, and thus improving wound healing. The quickening of wound closure is an important effect in the oral cavity to prevent bacterial infections and local or systemic chronic inflammation. 

**Figure 5:**
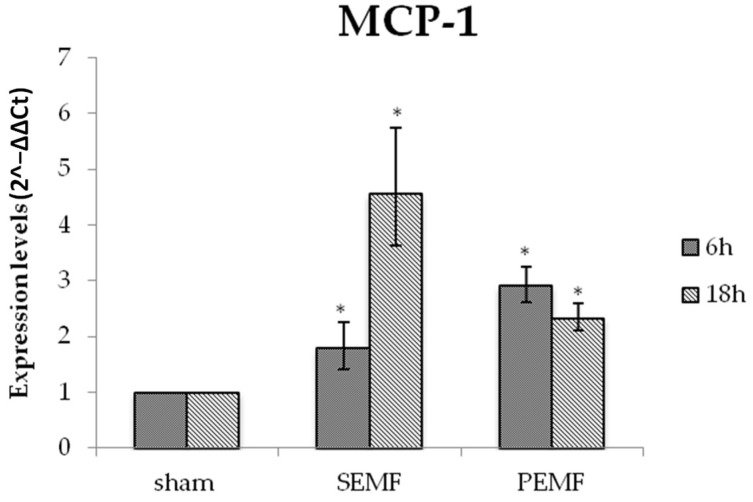
The mRNA expression of monocyte chemoattractant protein 1 (MCP-1) in hGFs exposed to the SEMF and the PEMF compared to sham-exposed cells. MCP-1 expression levels (2^−ΔΔ*C*t^) are reported as mean ± standard error (SE). * *p*-Value < 0.05 SEMF or PEMF vs. sham assumed as 1.

A different trend was observed for monocyte chemoattractant protein 1 (MCP-1) gene expression in exposed hGF cells. For SEMF exposure, the highest increase in MCP-1 expression (4.5 times higher than for sham exposure) was observed after 18 h, while for PEMF exposure, the highest increase in MCP-1 expression (3 times higher) was observed after 6 h, and a weaker increase (about 2.5 times higher) was observed after 18 h (Figure 5). These results are interesting since MCP-1 plays a key role in cell proliferation and migration and may be induced by exposure to SEMF or PEMF, leading to improved injury recovery.

#### 2.4.2. Effect of EMF on Metalloproteinase 2 (MMP-2) Gene Expression

**Figure 6:**
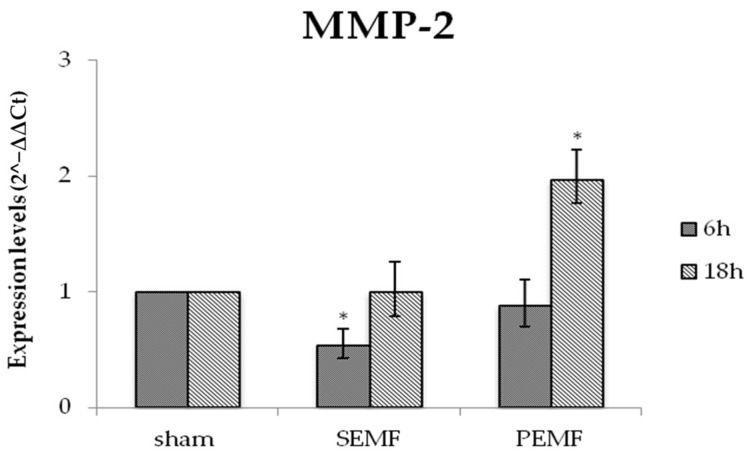
The mRNA expression of metalloproteinase 2 (MMP-2) in hGFs exposed to the SEMF and the PEMF compared to sham-exposed cells. MMP-2 expression levels (2^−ΔΔ*C*t^) are reported as mean ± standard error (SE). * *p*-Value < 0.05 SEMF or PEMF vs. sham assumed as 1.

Extracellular matrix remodeling and the induction of fibroblast migration were controlled by matrix metalloproteinase enzymes (MMPs). Among MMPs, we focused on metalloproteinase 2 (MMP-2), which has been reported to be associated with inflammation and matrix remodeling. Figure 6 reports changes in MMP-2 gene expression, showing a reduction in expression in hGFs after exposure to the SEMF for 6 h. Moreover, an increase in MMP-2 expression was detected after 18 h exposure to both the SEMF and the PEMF, respectively. Thus, exposure to each EMF type was responsible for a later increase in MMP-2 expression, although a higher increase was observed in hGF exposed to the PEMF. 

#### 2.4.3. Effect of EMFs on Inducible Nitric Oxide Synthase (iNOS) and Heme Oxygenase 1 (HO-1) Gene Expression

**Figure 7:**
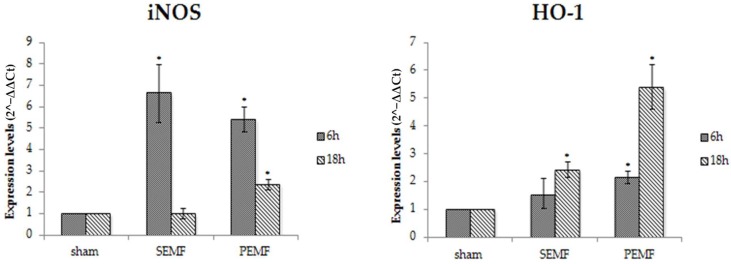
The mRNA expression of inducible nitric oxide synthase (iNOS) and heme oxygenase 1 (HO-1) in hGF exposed to the SEMF and the PEMF compared to sham-exposed cells. iNOS and HO-1 expression levels (2^−ΔΔCt^) are reported as mean ± standard error (SE). * *p*-Value < 0.05 SEMF or PEMF vs. sham assumed as 1.

Cell proliferation, collagen synthesis, and matrix remodeling are all key functions of fibroblasts, and play critical roles in successful wound healing. The activation of inducible nitric oxide synthase (iNOS) leads to the production of nitric oxide (NO), causing oxidative injury. The induction of the heme-catabolizing enzyme heme oxygenase 1 (HO-1) is important for modulating the activity and expression of iNOS. The mRNA expression levels of iNOS and HO-1 were evaluated in hGF after 6 and 18 h exposure to the SEMF or the PEMF. For both types of EMF, a significant increase in iNOS expression was detected after the 6 h exposure, while a reduction in expression was reported after the 18 h exposure, of about seven- and three-fold compared to the short (6 h) exposure for SEMF and PEMF exposure, respectively. However, different effects were observed for HO-1 gene expression. A weak increase after 6 h and a greater increase after 18 h of exposure were observed in both SEMF- and PEMF-exposed cells (Figure 7). Considering the anti-inflammatory and antioxidant properties of HO-1, and its ability to influence cell proliferation and migration, the increased expression of this enzyme after exposure to SEMF and PEMF might be able to quicken the wound-healing process.

## 3. Discussion

The restoration of the integrity of the gingival structure after injury is a complex process requiring the rapid healing and recovery of functions with minimal scarring to prevent the invasion of microbes or other agents deeper into tissues, thereby avoiding the development of chronic infection [44]. Gingival fibroblasts are the principal cell type involved in oral wound healing [2,45]. Several studies comparing fetal, gingival, and dermal fibroblasts have indicated that adult gingival fibroblasts share many properties with fetal fibroblasts, including their growth and migration properties, cell morphology, and cytokine production. The specific phenotype of gingival fibroblasts presents a higher expression of molecules involved in the regulation of inflammation and extracellular matrix remodeling [46,47]. This may in part underlie the ability of gingival wounds to heal faster with less scar formation compared to skin wounds. However, the relationship between the gingival fibroblast phenotype and the mechanisms leading to gingival wound healing has not yet been fully explored and clarified. Cell migration is a critical biological response during wound healing [48], and inflammatory mediators play a critical role in wound healing, being related to cell proliferation and migration and also to host defense against pathogens. 

Our previous studies evaluated the effects of SEMF exposure on keratinocytes in an in vitro model of skin wound repair. The ability of SEMF to activate the molecular and cellular machinery essential for rapid wound repair was highlighted [43]. The potential use of ELF-EMF to promote the wound-healing process following oral injuries may represent a novel frontier in the health fields, with an interesting clinical perspective. The reduction of wound healing time may be important to decrease the accumulation of bacteria, and ELF-EMF exposure, through the stimulation of growth factors and cytokine production, creates an appropriate environment to facilitate tissue regeneration [49,50]. 

In this study, we investigated the effects of a 1 mT ELF SEMF and a 1 mT ELF PEMF on hGFs in a model of wound healing. Our results show that exposure to each kind of ELF-EMF leads to increased cell proliferation. This observation is in accordance with observations by Dogru et al. in gingival tissue from albino rats exposed to SEMF and PEMF [2], and also with our previous observations of the human keratinocytes cell line [51]. The increased cell proliferation and migration induced by the SEMF and the PEMF were both responsible for improved wound closure, and a weaker cellular proliferation-promoting effect was observed in the presence of mitomycin C. The mechanisms by which SEMF and PEMF can promote wound healing are related to cell proliferation and migration and to the earlier peak of the expression of pro-inflammatory cytokines. IL-6 is a pleiotropic cytokine involved in the growth and differentiation of different cell types and in the up-regulation of MMP-1 in hGFs [52]. TGF-β is a family of multifunctional 25 kDa proteins (TGF-β1, 2, and 3) that is released by platelets, monocytes/macrophages, endothelial cells, and fibroblasts, all of which are essential cells for the wound repair process. TGF-β regulates the differentiation of endothelial cells, fibroblasts and immune cells, inducing chemotaxis of inflammatory cells, promoting the healing of soft and hard tissue, and influencing the inflammatory response, angiogenesis, the formation of granulation tissue, re-epithelization, and remodeling [53]. Chemokines are small chemotactic cytokines responsible for the directional migration of immune cells, increased cell proliferation, and modulating the release of inflammatory cytokines, thus regulating the wound-healing process [54]. During the inflammatory process in oral mucosal tissues, a higher concentration of MCP-1, produced by T cells and fibroblasts, actively aids the repair process. Fibroblast proliferation and the migration of macrophages and immune cells are induced by the inflammatory process in oral mucosal tissue, potentially initiating an autologous feedback loop that stimulates the wound-healing environment. 

In this study, we observed that levels of IL-6, TGF-β, and MCP-1 peaked after 6 h of exposure to each kind of ELF-EMF, with a subsequent decline in levels observed after 18 h of exposure. Previous studies have demonstrated that the duration of the inflammatory phase and persistently high concentrations of inflammatory mediators can impair, or at least delay, oral healing. Thus, the timing of the expression of inflammatory cytokines during the healing process driven by ELF-EMF exposure leads to the transition from the inflammatory to the reparative phase of wound healing. MCP-1 expression levels progressively increase over time, in accordance with an increased fibroblast proliferation rate and faster healing. These results are not in line with data obtained by Vianale et al., which show that human keratinocytes (HaCaT cell line) showed an early down-expression of MCP-1 mRNA after exposure to a 1 mT EMF [55]. These differences in results may be due to the different functional property of HaCaT cells and to the cell-related specificity of the biological effects of EMFs, as reported by Sul et al., who highlighted the cell type-specific response and disparity in sensitivity of different tissues to EMF exposure [56]. MMP-2, which is expressed by activated fibroblasts, is involved in matrix turnover, cell migration, and the modulation of the production of growth factors. The role of MMP-2 expression in phenotypic fibroblast transitions associated with wound healing has not been completely clarified. In the present study, we observed that, for both kinds of EMF, MMP-2 expression levels were down-regulated in cells exposed to the shorter EMF duration (6 h) and were up-regulated after 18 h of exposure, compared with sham-exposed cells. Our results show that exposure to each EMF type strengthens the inverse relationship between the levels of the inflammatory cytokines IL-6 and TGF-β, and the levels of MMP-2. Thus, the modulation of MMP-2 expression by EMFs may represent a fail-safe mechanism to delay the extracellular matrix turnover until fibroblasts are committed to a migratory phenotype. 

Although at low concentrations reactive oxygen species (ROS) can accelerate the wound repair process, at higher concentrations they may be responsible for tissue damage related to chemokines and inflammatory mediators. Patruno et al. found that the exposure of HaCaT cells to ELF-EMF increased expression levels of iNOS and endothelial nitric oxide synthase (eNOS), related to increased NOS activity and production [42]. Therefore, we examined the iNOS gene expression in hGFs exposed to the SEMF and the PEMF during wound healing. In both SEMF- and PEMF-exposed hGFs, iNOS expression increased significantly after 6 h of exposure, while after longer exposure times a slow decline in expression was observed in PEMF-exposed cells and a significant reduction in expression was observed in SEMF-exposed cells, relative to the expression levels detected in sham-exposed cells. The down-regulation of iNOS may be related to the reduced expression of TGF-β and IL-6 induced by SEMF and PEMF exposure. HO-1 is highly inducible in response to various stimuli such as oxidative stress and inflammation. In a previous study, we observed a significantly higher mRNA and protein expression of HO-1 in neuronal-like SH-SY5Y neuroblastoma cells after a short exposure to a SEMF, while longer exposures did not significantly affect HO-1 expression [57]. We showed that HO-1 expression levels were progressively increased in SEMF- and PEMF-exposed hGFs, and hypothesized that the induction of increased HO-1 expression might contribute to the enhancement of wound healing. These observations are in accordance with the findings of Grochot-Przeczek et al., who related the major HO-1 expression and the major HO-1 expression to accelerated scratch closure in a monolayer of primary transgenic keratinocytes [58]. During wound healing, exposure to PEMF or SEMF causes a counter-regulation of NO synthesis, which reduces iNOS activity and increases HO-1 expression. The increased iNOS expression in ELF-EMF-exposed cells compared with sham-exposed cells observed in the present study confirmed that EMF exposure leads to early oxidative stress and then to an antioxidant protective mechanism with an increase of HO-1, a protective agent against oxidative and inflammatory insults. These results are also consistent with those of our previous studies which showed an increase of catalase levels to counteract the rise in NOS activity and O_2_ levels in ELF-EMF-exposed SH-SY5Y [59]. As already suggested, the pharmacological induction of HO-1 is associated with a significant acceleration of wound healing and the attenuation of the inflammatory response in the wounded corneal epithelium [60] and, therefore, we suggest an association between the induction of HO-1 expression by SEMF and PEMF and the reduction of pro-inflammatory molecules. A delay in tissue repair due to a decreased capacity of proliferative or migratory cells negatively affects the aesthetics and functioning of osseo-integrated oral implants, especially in surgically installed implants, leading to an unattractive condition and an increased rehabilitation time. In diabetic patients, wound healing after dental treatment is impaired, with defective fibroblast migration and proliferation [61]. Thus, in vivo EMF treatment may be an alternative and promising approach in the treatment of diabetic patients. 

Furthermore, considering the cellular interactions of gingival fibroblasts with immune cells, and their possible role in the proliferation of immune cells [62], the ability of EMF exposure to modulate cytokines and chemokines in hGFs could represent a new way of orchestrating modulatory events for gingival tissue homeostasis. 

In summary, our study indicates that exposure to an SEMF accelerates wound healing, probably by inducing an early increase of hGF proliferation and the expression of the inflammatory mediators IL-6, TGF-β, and iNOS, thus driving the shift from the inflammatory to the reparative phase of wound healing. Moreover, exposure to a PEMF improves the wound area closure rate compared to sham-exposed hGFs, with a better effect observed after 18 h of exposure. These results are encouraging, and show that the application of ELF-EMF is a nontoxic way to provide a good environment for the healing of oral tissue. Such applications could be promising in regenerative medicine and allow new non-pharmacological strategies for tissue repair. However, additional molecular and clinical studies are needed to elucidate the best healing timeline, and the best shape and frequency of ELF-EMF, for treating gingival injuries. The development of non-invasive EMF devices for non-traditional therapies could represent a goal to be achieved in order to reduce patient pain and gingival healing time.

## 4. Materials and Methods

### 4.1. Collection of Tissue Samples

Gingival tissue biopsies for primary hGF isolation and culturing were obtained from 11 healthy human subjects, aged between 45 and 75 years, who were recruited as patients at the Dental Clinic of D’Annunzio University, Chieti, Italy. The subjects provided written informed consent for the use of discarded tissues removed from an extracted tooth, for research purposes. Each sample was coded to guarantee the anonymity of the donors. The gingival tissues were placed in a tube with physiological solution, at room temperature, for transport to the cell culture laboratory. The study was approved on 23 July 2015 by the Inter-Institutional Ethic Committee of the University of Chieti–Pescara, Chieti, Italy; committee report no. 14.

### 4.2. Primary Human Gingival Fibroblast Cell Culture

Gingival tissue biopsies were derived from irregularly shaped samples and most tissue specimens were 6 × 2 mm. Tissue were washed with phosphate buffered saline (PBS), and placed in a T-25 flask with a surface area of 25 cm^2^ (Euroclone) for the culturing; the flasks were flooded with Dulbecco’s Modified Eagle’s Medium (DMEM) (pH 7.2; Merck KGaA, Darmstadt, Germany) supplemented with 10% heat-inactivated fetal bovine serum (FBS), 100 U/mL penicillin, 100 μg/mL streptomycin, and 2 mmol/L l-glutamine (Merck KGaA), and were then placed in a humidified incubator with 5% CO_2_ and 95% air at a temperature of 37 °C. The culture media were replaced with fresh medium twice a week. After reaching 70–80% confluence by the primary cells, hGFs were detached with 0.05% trypsin-ethylenediaminetetraacetic acid (trypsin-EDTA) (Merck KGaA) and then sub-cultured at a ratio of 1:4. hGFs were used from the third through fifth passages. 

### 4.3. Generation and Application of Magnetic Fields

**Figure 8:**
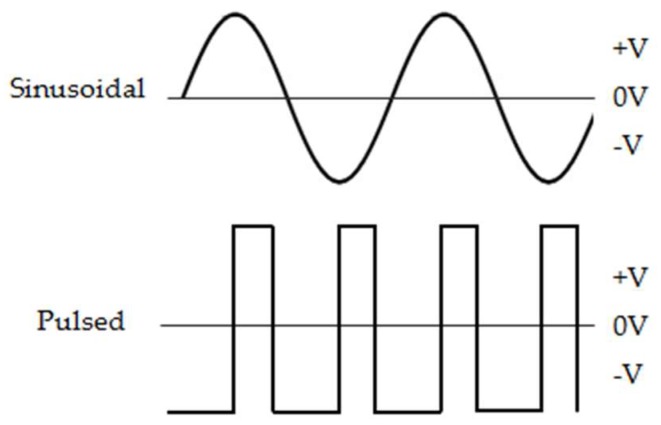
Schematic representation of the waveforms of the extremely low-frequency EMFs. PEMF parameters were 0.3 ms width duration, 12 Hz frequency, and 142 mV curve amplitude, SEMF parameters were 50 Hz frequency and 42 mV curve amplitude, and both EMFs had a flux density of 1 mT (rms).

The ELF-EMFs were generated using a system producing an oscillating magnetic field (AC/MF) consisting of (1) a 33220A signal generator (Agilent Technologies, Santa Clara, CA, USA), (2) an NAD 216 power amplifier (NAD Electronics, Pickering, ON, Canada), (3) an ISR 658 oscilloscope (Iso-Tech, Vicenza, Italy) dedicated to the monitoring of output signals from an MG-3D Gaussmeter (Walker Scientific Inc., Worcester, MA, USA) and the 33220A signal generator, and (4) a device composed of a 160-turn solenoid (22 cm length, 6 cm radius, 1.25 × 10^−5^ cm copper wire diameter). The ELF-EMF intensity established was 1 mT (rms), and the field strength and distribution were measured with a Hall-effect probe connected to an MG-3D Gaussmeter. For the application of the PEMF, the applied magnetic field consisted of pulse bursts with a width duration of 1.3 ms repeated at a rate of 12 Hz using a voltage of 168 mV. For the sinusoidal SEMF at a flux density of 1 mT (rms), the signal generator was set to the sine wave setting with a frequency of 50 Hz and a wave amplitude of 42 mV (Figure 8).

### 4.4. EMF Exposure Procedures

The solenoid was placed into a cell incubator with 5% CO_2_ and 95% air at 37 °C, with its center ventilated by the fan for appropriate air circulation. Due to the Joule effect, heat is dispersed by the continuous forced ventilation in the total mass of the CO_2_ incubator. In order to study the effects of the SEMF and PEMF on mechanisms of wound healing, the hGFs obtained from each subject were placed in the middle of the solenoid, on a transparent polymethylmethacrylate holder. Temperature was continuously recorded by an HI 9274 OC printing thermometer (Hanna Instruments, Woonsocket, RI, USA), placed in the center of the solenoid; measured temperatures were in the range of 37 ± 0.3 °C. The cells in the sham-exposed group, which served as a control, were placed in the current-less Helmholtz coils. For confirmation, cells were also placed in another cell incubator without an EMF, with the same temperature and CO_2_ conditions as the sham-exposed cells. No differences were detected with respect to the sham-exposed cells. All experiments were conducted under blind conditions.

### 4.5. Cell Count and Viability

To assess the total number of cells, 0.2 × 10^6^ cells/mL of hGFs were placed in six well plates (Euroclone), in duplicate, for the wound-healing assay. The medium was removed and replaced by 0.25% trypsin/0.1% EDTA 1× solution (Merck KGaA) for 2 min to promote cell detachment from the acrylic substrate. In order to stop trypsin activity, the same amount of complete medium was added, and cells were collected and centrifuged at 1200× *g* for 5 min in order to obtain a pellet. The pellet was resuspended in 2 mL of DMEM and cells were counted using a Bürker chamber and a DM IL inverted light microscope (Leica Camera AG, Wetzlar, Germany). The number of cells obtained in the counting corresponds to the number of cells per milliliter of suspension. Cell viability was evaluated by Trypan blue dye exclusion. A total of 90 μL of 0.04% Trypan blue dye (Sigma-Aldrich Corp., St. Louis, MO, USA) was added to 10 μL of cell suspension and examined to determine the number of viable cells. The total number of living cells in the culture amounted to over 98% of viable cells, as determined by the exclusion of the blue Trypan dye.

### 4.6. In Vitro Mechanical Injury Model and Wound-Healing Assay

The scratch injury is a well-developed method to investigate in vitro cell migration during wound healing. The hGFs were seeded at a density of 0.2 × 10^6^ cells/well in six-well plates in the presence of complete medium and incubated at 37 °C and 5% CO_2_ (*v*/*v*) to almost confluence. After 24 h, a scratch (2–3 mm in size) was made on the cell monolayer by scraping the dish surface with a sterile tip (Ø = 0.1 mm), creating a uniformly sized scratch through the removal of cells from the scraped area. Subsequently, the rate at which cells divided and migrated into the wound area (i.e., ‘wound fill rate’) was determined microscopically using a DM IL inverted light microscope at a magnification of 10× and photographed with a Color View II digital camera (Leica Camera AG, Wetzlar, Germany). To measure the remaining cell-free area over time, experiments were carried out in triplicate wells, and hGFs derived from all recruited patients were analyzed. 

### 4.7. Cell Metabolism (MTT Uptake)

To evaluate cell metabolism, the MTT assay was used. This test is based on the cellular uptake of the yellow MTT compound that is reduced to formazan by mitochondrial nicotinamide adenine dinucleotide phosphate (NAD(P)H) oxidoreductases in viable cells, resulting in an intracellular insoluble blue product. Briefly, 4 × 10^3^/100 µL of hGFs were plated in 96 well plates (Euroclone) after confluence cells were exposed to the SEMF, PEMF, or sham for 6 and 18 h. Two hours before the fixed time points, a solution of 0.5 mg/mL of MTT was added to the culture medium, and at the end of the incubation cell-free supernatant was removed and cells were washed with 0.01 M of PBS (pH 7.4). In a different set of plates, mitomycin C (5 μg/mL) was added to the medium in order to inhibit cell proliferation, and was replaced with fresh medium after 2 h. Subsequently, 100 µL of dimethyl sulfoxide (DMSO) was added for 15 min in order to lyse cells and solubilize the produced blue insoluble formazan. The absorbance of each sample was measured at 550 nm using a Glomax Multidetection System (Promega, Milan, Italy). Colour intensity is proportional to the activity of mitochondrial dehydrogenases of living cells.

### 4.8. RNA Extraction, Retro-Transcription and Real-Time Polymerase Chain Reaction

Total RNA was extracted from hGFs collected after the wound-healing assay, using QIAzol reagent (Qiagen, Hilden, Germany) according to the manufacturer’s protocol. The RNA concentration was determined by measuring the samples’ absorbance at 260 nm using a NanoDrop 2000 ultraviolet-visible (UV-Vis) spectrophotometer (Thermo Fisher Scientific, Waltham, MA, USA), and its purity was assessed by the absorbance ratios at 260/280 and 260/230 nm. For each sample, 1 μg of mRNA was reverse-transcribed into complementary DNA (cDNA) using a QuantiTect Reverse Transcription Kit (Qiagen). Subsequently, a real-time polymerase chain reaction (PCR) was performed using the GoTaq^®^ qPCR Master Mix (Promega, Madison, WI, USA) to evaluate the gene expression of IL-6, TGF-β, MCP-1, MMP-2, iNOS, HO-1, and 18s housekeeping gene (Table 1). All PCR reactions were performed in triplicate in a MasterCycler EP Thermal Cycler (Eppendorf, Hamburg, Germany) with the following conditions: initially, 2 min incubation at 95 °C followed by 40 cycles consisting of 30 s at 95 °C, then 1 min at 60 °C, and 30 s at 68 °C. The analysis of the melting curve was performed in the temperature range of 60 to 95 °C at the end of each run. Gene expression analysis was performed according to the ΔΔ*C*_t_ method, in which the relative expression of each gene was at first normalized by the housekeeping gene (18 s), obtaining Δ*C*_t_ for the experimental sample and for the calibrator sample, where Δ*C*_t_ = *C*_t_ target gene – *C*_t_ housekeeping gene. Relative fold changes in gene expression were determined by the 2^−ΔΔ*C*t^ method, where ΔΔ*C*_t_ = Δ*C*_t_ experimental sample −Δ*C*_t_ calibrator sample.

### 4.9. Statistical Analysis

Results concerning cell viability and metabolism were expressed as mean ± SD, and were obtained from hGFs isolated from all recruited patients, analyzed in duplicate. The percentage of cell-free area in the wound-healing assay was obtained from the measurements, in centimeters, made for each hGF primary line, and were expressed as relative values with respect to sham exposure at baseline (0 h). To provide an accuracy assessment of fold change, calculated with the 2^−ΔΔ*C*t^ method, the 95% confidence interval (95% CI) and standard error (SE) were determined. A Student’s *t*-test for paired data was applied to evaluate the significance of the difference in gene expression levels over time, while a Student’s *t*-test for unpaired data was applied to evaluate the significance of differences in the expression levels of mRNA cytokines between SEMF and sham-exposed cells and between PEMF and sham-exposed cells. In all statistical tests, the threshold of statistical significance was assumed to be equal to *p* ≤ 0.05. Data were analyzed using the SPSS^®^ Advanced Statistical TM 13 software (Chicago, IL, USA) and the R open-source software. 

## Figures and Tables

**Table 1:** Primer sequences. 18s: housekeeping gene 18s; TGF-β: transforming growth factor beta 1; IL-6: Interleukin-6; MCP-1: monocyte chemoattractant protein 1; iNOS: inducible nitric oxide synthase; HO-1: heme oxygenase; MMP-2: matrix metalloproteinase-2.

Gene	Forward Primer Sequence (5′–3′)	Reverse Primer Sequence (5′–3′)
18s	CTTGCCATCACTGCCATTAAG	TCCATCCTTTACATCCTTCTGTC
TGF-b	AACAATTCCTGGCGATACCTC	GATGTGAACCCGTTGATGTCC
IL-6	GTACATCCTCGACGGCATC	ACCTCAAACTCCAAAAGACCAG
MCP-1	GGTCCCTGTCATGCTTCTGG	CCTGCTGCTGGTGATCCTCT
iNOS	GGTATCCTGGAGCGAGTGGT	CTCTCAGGCTCTTCTGTGGC
HO-1	TCTTCACCTTCCCCAACATTG	CTCTGGTCCTTGGTGTCATG
MMP-2	CAGTGACGGAAAGATGTGGT	TGGTGTAGGTGTAAATGGGTG

## Abbreviations

EMF	Electromagnetic fields
LF	Low frequency
ELF	Extremely low-frequency
hSSCs	Human skeletal stem cells
hBMSCs	Human bone marrow stromal cells
hGF	Human gingival fibroblasts
PBS	Phosphate buffered saline
DMEM	Dulbecco’s Modified Eagle’s Medium
EDTA	Ethylenediaminetetraacetic acid
PEMF	Pulsed electromagnetic fields
SEMF	Sinusoidal extremely low-frequency electromagnetic field
Hz	Hertz
mT	Millitelsa
mV	Millivolt
MTT	3-(4,5-Dimethylthiazol-2-yl)-2,5-diphenyl tetrazolium bromide
DMSO	Dimethyl sulfoxide
mRNA	Mitochondrial RNA
cDNA	Complementary DNA
IL-6	Interleukin-6
TGF-β	Transforming growth factor beta 1
MCP-1	Monocyte chemoattractant protein 1
MMP-2	Matrix metalloproteinase-2
TNF-α	Tumor necrosis factor alpha
ROS	Reactive oxygen species
iNOS	Inducible nitric oxide synthase
eNOS	Endothelial nitric oxide synthase
HO-1	Heme oxygenase 1
